# Detailed analysis of the pathologic hallmarks of Nipah virus (Malaysia) disease in the African green monkey infected by the intratracheal route

**DOI:** 10.1371/journal.pone.0263834

**Published:** 2022-02-10

**Authors:** Curtis Cline, Todd M. Bell, Paul Facemire, Xiankun Zeng, Thomas Briese, W. Ian Lipkin, Joshua D. Shamblin, Heather L. Esham, Ginger C. Donnelly, Joshua C. Johnson, Lisa E. Hensley, Anna N. Honko, Sara C. Johnston

**Affiliations:** 1 Pathology Division, United States Army Medical Research Institute of Infectious Diseases, Fort Detrick, Maryland, United States of America; 2 Center for Infection and Immunity, Columbia University Mailman School of Public Health, New York, New York, United States of America; 3 Veterinary Medicine Division, United States Army Medical Research Institute of Infectious Diseases, Fort Detrick, Maryland, United States of America; 4 Virology Division, United States Army Medical Research Institute of Infectious Diseases, Fort Detrick, Maryland, United States of America; 5 Infectious Disease Research Portfolio, Strategy & Operations, Moderna, Boston, Massachusetts, United States of America; 6 Office of the Chief Scientist, National Institute of Allergy and Infectious Disease Integrated Research Facility, Fort Detrick, Maryland, United States of America; 7 Nonclinical Studies Unit, Boston University School of Medicine National Emerging Infectious Diseases Laboratories, Boston, Massachusetts, United States of America; Lewis Katz School of Medicine, Temple University, UNITED STATES

## Abstract

Disease associated with Nipah virus infection causes a devastating and often fatal spectrum of syndromes predominated by both respiratory and neurologic conditions. Additionally, neurologic sequelae may manifest months to years later after virus exposure or apparent recovery. In the two decades since this disease emerged, much work has been completed in an attempt to understand the pathogenesis and facilitate development of medical countermeasures. Here we provide detailed organ system-specific pathologic findings following exposure of four African green monkeys to 2.41×10^5^ pfu of the Malaysian strain of Nipah virus. Our results further substantiate the African green monkey as a model of human Nipah virus disease, by demonstrating both the respiratory and neurologic components of disease. Additionally, we demonstrate that a chronic phase of disease exists in this model, that may provide an important opportunity to study the enigmatic late onset and relapse encephalitis as it is described in human disease.

## Introduction

Nipah virus (NiV) is a zoonotic paramyxovirus within the genus *Henipavirus* that can infect and cause disease in a variety of mammalian hosts including pigs, cows, cats, dogs, sheep, goats, horses and humans. Fruit bats (also referred to as flying foxes) are considered to be the major reservoir, with pigs occasionally acting as intermediate hosts [1, 2]. The disease has been either associated with naturally occurring outbreaks and/or reported in reservoir species throughout countries in Southeast Asia including Malaysia, Cambodia, Thailand, Singapore, Bangladesh and India, among others, with sporadic outbreaks occurring since the disease was first reported in Malaysia in 1998. Characteristics that may contribute to the ability of NiV to cause a pandemic have also been identified, including the potential for a high mutation rate, its presence in areas with high population density, a respiratory component to the disease, human susceptibility and potential for human-to-human transmission with some strains [3]. Two predominant clades of the virus have been identified as Nipah-Malaysia (NiV-MY, Malaysia and Cambodia) and Nipah-Bangladesh (NiV-BD, Bangladesh and India) [1]. Transmission and fatality rates vary geographically due to pathogenic differences between circulating isolates, with fatality rates in Bangladesh and India being reported as higher (above 90% in some outbreaks, compared to just above 30% in Malaysia) [4, 5]. Additionally, studies utilizing the African green monkey (AGM) model have reported differences in pathology and higher lethality in NiV-BD compared to NiV-MY [6, 7]. NiV infection in humans generally occurs by ingestion of foods that contain the virus (i.e. fresh or fermented date palm sap or undercooked infected pork), by contact with infected human or animal body fluids where droplet transmission may occur, or by close contact with pigs [1]. Transmission between humans has been reported in some outbreaks in Bangladesh [1], most often in healthcare settings or due to contact with an infected corpse [8]. The more prominent respiratory component of disease caused by NiV-BD isolates may result in an increase in transmissibility and the aforementioned human-to-human transmission [9].

The main clinical presentation of NiV disease is both respiratory and neurologic [10, 11]. Patients initially present with a fever and altered mental status that may progress to coma and death. Survivors may have persistent, or relapsing CNS disease characterized by the onset of new neurologic signs after recovery from acute infection [12–14].

Gross lesions in cases of NiV infection in humans have been described as non-specific but rarely may include hemorrhagic or necrotic lesions in the central nervous system (CNS) [15]. A hallmark pathological finding for fatal NiV cases is CNS vasculitis in the brain accompanied by thrombosis and perivascular cuffing. Vasculitis is also reported systemically in other organs such as the heart and kidneys. Additional lesions in the CNS may include necrosis and thrombosis [15]. Pneumonia, fibrinoid necrosis and vasculitis are reported in the lungs. Syncytial cells may form from vascular endothelium, and [1, 15] lymphoid depletion and necrotizing lesions are reported in the spleen [15].

Various animal species have been investigated pursuant to finding a model for studying NiV pathogenesis, as well as finding an appropriate model to evaluate therapeutic and prophylactic interventions. These include guinea pigs, pigs, hamsters, cats, ferrets, AGM and squirrel monkeys [16]. Of these animal models, the AGM is characterized as having valuable pathologic features that are relevant to human NiV disease including the presence of both neurologic and respiratory disease manifestations, as well as systemic vascular infection [16–18]. Because this model recapitulates respiratory disease, neurologic disease, and vasculitis, it is relevant for studying disease pathogenesis and countermeasure efficacy. Additionally, magnetic resonance imaging findings in infected AGM closely correlate with findings in cases of human infection [17].

Although numerous studies have been conducted using the AGM NiV model, further characterization of this model to define critical endpoints for efficacy evaluations is still necessary. Current strategies for inoculating AGM with NiV include intratracheal (i.t.) or aerosol (a.e.) exposure [18–20], nasal mucosal atomization (n.m.a) [21], or exposure using a combination of the i.t. and intranasal (i.n.) routes [6, 7]. From a natural infection perspective, the i.t./i.n. and a.e. inoculation strategies are clinically relevant as they mimic exposure to an infectious aerosol; however, these models are not yet well characterized as only a limited number of studies have been performed.

The i.t. NiV-MY AGM model has been better characterized to date, and has been used for countermeasure evaluations. Additionally, some studies have demonstrated survivors using this model, which may provide an opportunity to study late onset and recurrent neurologic manifestations of this disease [17, 18]. The goal of the present study was to further characterize pathologic changes associated with the NiV-MY AGM i.t. model, and define how these changes relate to human clinical disease. Four AGM were infected via the i.t. route with 2.41×10^5^ pfu of NiV-MY; a bronchoscope MicroSprayer^®^ assembly was used for administration to allow challenge inoculum placement verification. Use of a microsprayer generates particles that are approximately 16–22 μM in diameter; this is slightly larger than the range in droplet size in a human sneeze (0.5–12 μM) [22, 23] or cough (0.62–15.9 μM) [24]. All four animals either succumbed or met euthanasia criteria prior to the end of the study. Pathologic and histopathologic lesions for the three acute phase animals were consistent with systemic NiV disease, and one animal that survived the acute NiV infection phase went on to develop chronic neurologic disease similar to that described by Johnston *et al*. [18]. The results confirm that encephalitis described for human cases can be replicated using this model, and the presence of a chronic phase of disease (as described by Johnston *et al*.) may provide an opportunity to study the disease’s unique neurologic manifestations.

## Materials and methods

### Animals

Animal research was conducted at the United States Army Medical Research Institute of Infectious Diseases (USAMRIID). Four adult, male, henipavirus-naïve *Chlorocebus aethiops* (African Green monkeys; AGM) of Caribbean origin were used on this study and the source was the USAMRIID colony. Because AGM are wild caught, the exact ages of subjects are not known, but based on the time in the colony it is assumed that these animals were at least 3 years old at time of challenge. All animals used on this study were male, and henipavirus serologically naïve. All animals had passed a semi-annual physical examination and were certified as healthy by a veterinarian. Animals were acclimated in ABSL-4 animal rooms for 7 days prior to virus exposure, and were individually housed in 4.4 square foot cages (27” L x 23.5” W x 34” H). Animals were provided 2050 Monkey Chow (Harlan Teklad, Frederick, MD), fruits, and water *ad libitum* via an automatic watering system, with other enrichment provided regularly as recommended by the Guide for the Care and Use of Laboratory Animals.

### Ethics statement

These experiments and procedures were reviewed and approved by the United States Army Medical Research Institute for Infectious Diseases Institutional Animal Care and Use Committee (IACUC). All research was conducted in compliance with the USDA Animal Welfare Act (PHS Policy) and other federal statutes and regulations relating to animals and experiments involving animals, and adheres to the principles stated in the Guide for the Care and Use of Laboratory Animals, National Research Council, 2011. The facility is fully accredited by the Association for Assessment and Accreditation of Laboratory Animal Care, International. The animals were provided food and water ad libitum and checked at least daily according to the protocol. All efforts were made to minimize painful procedures; the attending veterinarian was consulted regarding painful procedures, and animals were anesthetized prior to phlebotomy and virus infection. Following the development of clinical signs, animals were checked multiple times daily. When clinical observations and scores of animals reached defined levels based on the approved IACUC protocol, animals were euthanized under deep anesthesia to minimize pain and distress. Animals were humanely euthanized (when moribund or at the end of study) by intracardiac administration of a pentobarbital-based euthanasia solution under deep anesthesia in accordance with current American Veterinary Medical Association Guidelines on Euthanasia and institute standard operating procedures.

### Virus and virus exposure

The Malaysia strain of NiV used on this study has been described previously (Johnston et al. ref). On the day of challenge, post-infection day (PID) 0, a stock of NiV was prepared by making the required dilutions to achieve a target dose of 2.0×10^5^ pfu in MEM Alpha GlutaMax-I (Life Technologies, Grand Island, NY) containing 10% heat-inactivated fetal calf serum (Life Technologies, Grand Island, NY). A bronchoscope MicroSprayer^®^ assembly was used for virus administration. Animals were anesthetized using Telazol^®^ at a dose of approximately 4mg/kg. Briefly, the syringe of a Custom Length MicroSprayer^®^/Syringe Assembly Aerosolizer (Penn-Century^™^, Wyndmoor, PA) was filled with 1 mL of inoculum at a target dose of 2.0x10^5^ pfu/mL, and the tip of the MicroSprayer^®^ was passed through a bronchoscope to the tracheal carina. The bronchoscope used was a flexible optical fiber optic instrument attached to a monitor for real-time visualization of administration of the virus and uptake into both main stem bronchi.

### Animal observations and euthanasia

Animals were evaluated cage side for signs of illness. Other observations such as biscuit/fruit consumption, condition of stool, and urine output were also documented, if possible. Once clinical signs of illness were apparent, animals were observed up to three times daily, with no fewer than 6 hours between observations. More frequent observations (i.e., less than 6 hours apart) were conducted when deemed necessary by the PI or study veterinarian due to the critical nature of disease for one or more animals. Observations under anesthesia (i.e., ketamine administered at 10mg/kg for physical examinations) occurred after cage side observations on PID-8, -1, 0, 1, 3, 5, 7, 10, 12, 14, and the day of euthanasia, and included the collection of clinical disease sign data, body weights, rectal temperatures, and blood.

Animals were humanely euthanized when moribund by intracardiac administration of a pentobarbital-based euthanasia solution under deep anesthesia in accordance with current American Veterinary Medical Association Guidelines on Euthanasia and institute standard operating procedures. Anesthesia was induced using Telazol^®^ administered at approximately 6mg/kg. Observations for euthanasia criteria assessment included responsiveness (0 = normal, 1 = mild unresponsiveness but active when approached, 2 = moderate unresponsiveness and withdraws when approached, 3 = severe unresponsiveness and does not withdraw when approached, 4 = unresponsive with no pain response), recumbency (0 = normal, 1 = occasional prostration, 2 = persistent prostration but normal when approached, 3 = persistent prostration), respiration (0 = normal, 1 = mildly labored, 2 = labored, 8 = agonal), bleeding (0 = none, 1 = mild, 2 = moderate, 3 = severe/copious), and seizures (0 = none, 1 = mild/petit mal, 2 = moderate/tonic-clonic, 3 = severe/tonic-clonic with or without delayed recovery, 4 = continuous). If the total euthanasia criteria score was 8 or if responsiveness = 4 or seizures = 4, the animal was considered moribund and was euthanized.

### Clinical pathology

For serum chemistries, whole blood was collected into Z Serum Clot Activator Greiner Vacuette tubes (Greiner Bio-One, Monroe, NC). Tubes were allowed to clot for 30–60 min and the serum separated in a centrifuge set at 1800 × g for 10 min at ambient temperature. Serum was removed from the clot within 1 hour of centrifugation and was analyzed within 12 hours of collection. Chemistry analysis was performed on Piccolo Point-Of-Care Analyzers (Abaxis, Union City, CA) using a General Chemistry 13 panel (Abaxis, Union City, CA). Complete blood counts (i.e., hematology) were performed within 4 hours of collection on K3 EDTA whole blood using the Hemavet 950 FS instrument (Drew Scientific, Waterbury, CT).

### Peripheral blood mononuclear cell (PBMC) isolations

For PBMC isolations, 1.5 mL of K3 EDTA whole blood was added to 1.5 mL of phosphate buffered saline (w/o calcium or magnesium) (PBS-/-; Life Technologies, Grand Island, NY). PBMC isolations were performed as described previously [18]. Following isolation, 600 μL of TRI Reagent-LS (Sigma Aldrich, St. Louis, MO) was added for RNA extraction and qRT-PCR.

### RNA extraction and quantitative real-time polymerase chain reaction (qRT-PCR)

RNA extraction and qRT-PCR were performed on PBMCs as described previously [18]. Assays were run in duplicate and target copy numbers calculated based on Ct values in reference to serial dilutions of a calibrated plasmid standard containing the cloned target region.

### NiV plaque assay

Virus exposure dose titration was performed by Avicel plaque assay as described previously [18]. The average number of plaques in triplicate wells was multiplied by 5 to determine pfu/mL, and this value was multiplied by the dilution factor to determine the exposure dose in pfu/mL.

### VSV-NiV pseudotype neutralization assay

Multicycle VSV-pseudotype neutralization assays were performed as described previously [18], using a recombinant vesicular stomatitis virus (VSV) construct whose glycoprotein (G) gene was replaced by a red fluorescent protein reporter gene and that was pseudotyped on NiV G and F glycoprotein expressing cells through cotransfection with the respective individual plasmids. Neutralization was determined by reduction in red fluorescent protein reporter gene signal compared to the control (cells and pseudotyped virus alone, no test serum added).

### Gross necropsy

Necropsies were performed by a board-certified veterinary pathologist, and the following tissues were collected for histology and molecular pathology: samples of skin; trachea; esophagus; larynx; mandibular salivary gland; submandibular, mediastinal, axillary, inguinal, and mesenteric lymph nodes; testes; prostate; ovary; uterus; liver; spleen; adrenal glands; thyroid gland; parathyroid gland; pituitary gland; kidneys; urinary bladder; aorta; heart; lung; bone marrow; glandular stomach; pylorus; pancreas; duodenum; jejunum; ileum; cecum; colon; and brain. Tissues were fixed by immersion in 10% neutral buffered formalin (Valtech diagnostics, Zelienople PA) for a minimum of 21 days.

### Histopathology

The tissue samples were trimmed, routinely processed, and embedded in paraffin. Sections of the paraffin-embedded tissues were trimmed at 5–6 μm thickness for histology. Slides were then deparaffined, stained with hematoxylin and eosin (H&E), coverslipped, and labeled. A board-certified veterinary pathologist evaluated the H&E slides using a Nikon Eclipse 80i light microscope (Nikon Inc, Melville, NY).

### Immunohistochemistry (IHC), immunofluorescence analyses (IFA) and *in situ* hybridization (ISH)

IHC was performed as described previously [18] using an immunoperoxidase detection system (EnVision; Dako) and a polyclonal antibody (USAMRIID #1294) against NiV. Normal cynomolgus macaque spleen served as the negative control. The positive control was spleen from an AGM that had previously succumbed to experimental NiV infection. Normal rabbit IgG was used as the negative serum control for the control slides. A board-certified veterinary pathologist evaluated the H&E slides using a Nikon Eclipse 80i light microscope (Nikon Inc, Melville, NY).

In order to co-label virus infected cells in the lung by IFA, formalin-fixed paraffin embedded (FFPE) tissue sections were deparafinized using xylene and a series of ethanol washes. After a 0.1% Sudan black B (Millipore Sigma, Temecula, CA) treatment to eliminate background autofluorescence, the sections were heated in Tris-EDTA buffer (10mM Tris Base, 1mM EDTA Solution, 0.05% Tween 20, pH 9.0) for 15 minutes to reverse formaldehyde crosslinks. After rinses with PBS (pH 7.4), the sections were blocked with PBT (PBS +0.1% Tween-20) containing 5% normal goat serum overnight at 4°C. The sections were then incubated with rabbit polyclonal antibodies against NiV (USAMRIID, #1294) at a dilution of 1:1,000 and mouse anti-human CD68 antibody (Clone KP1, Dako Agilent Pathology Solutions, Carpinteria, CA) at a dilution of 1:200, mouse anti-E-cadherin antibody (33–4000, Thermo Fisher Scientific) at a dilution 1:100, or mouse anti-Von Willebrand Factor (VWF, ab201336, Abcam, Cambridge, MA) at a dilution 1:100. After rinsing in PBT, sections were incubated with goat IgG Alexa Fluor 561-conjugated anti-rabbit and anti-mouse, and goat IgG Alexa Fluor 488-conjugated anti-mouse antibodies (Thermo Fisher Scientific) for 1 hour at ambient temperature. Sections were cover-slipped using VECTASHIELD antifade mounting medium with DAPI (Vector Laboratories, Burlingame, CA). Images were captured on a Zeiss LSM 880 confocal system (Zeiss, Oberkochen, Germany) and processed using ImageJ software (National Institutes of Health, Bethesda, MD).

To detect NiV genomic RNA in FFPE tissues, ISH was performed using the RNAscope 2.5 HD RED kit (Advanced Cell Diagnostics, Newark, CA) according to the manufacturer’s instructions. Briefly, an ISH probe targeting NiV genome (nucleotides 2,400–4,400, GenBank #JN808857.1) was designed and synthesized by Advanced Cell Diagnostics (Cat# 520071). Tissue sections underwent deparaffinization with xyless II (Valtech, Brackenridge, PA) and a series of ethanol washes and peroxidase blocking. The sections were then heated in kit-provided antigen retrieval buffer and digested by kit-provided proteinase. Sections were exposed to ISH target probe pairs and incubated at 40°C in a hybridization oven for 2 hours. After rinsing with wash buffer, ISH signal was amplified using kit-provided Pre-amplifier and Amplifier conjugated to alkaline phosphatase, and sections were incubated with Fast Red substrate solution for 8 minutes at ambient temperature. Sections were then stained with hematoxylin, air-dried, and mounted.

## Results

### Detailed pathological examination of the i.t. NiV-MY AGM model

A manuscript published by Johnston *et al*. in 2015 [18] provided a detailed overview of the disease caused by NiV-MY when AGM are infected by the i.t. route. To more extensively study the pathological features associated with NiV disease in AGMs and attempt to replicate the chronic-phase neurological disease described in the initial study, a follow on study was performed in which four additional adult AGM were exposed to 2.41×10^5^ pfu of NiV-MY by the i.t. route using a bronchoscope MicroSprayer^®^ assembly.

**Fig 1:**
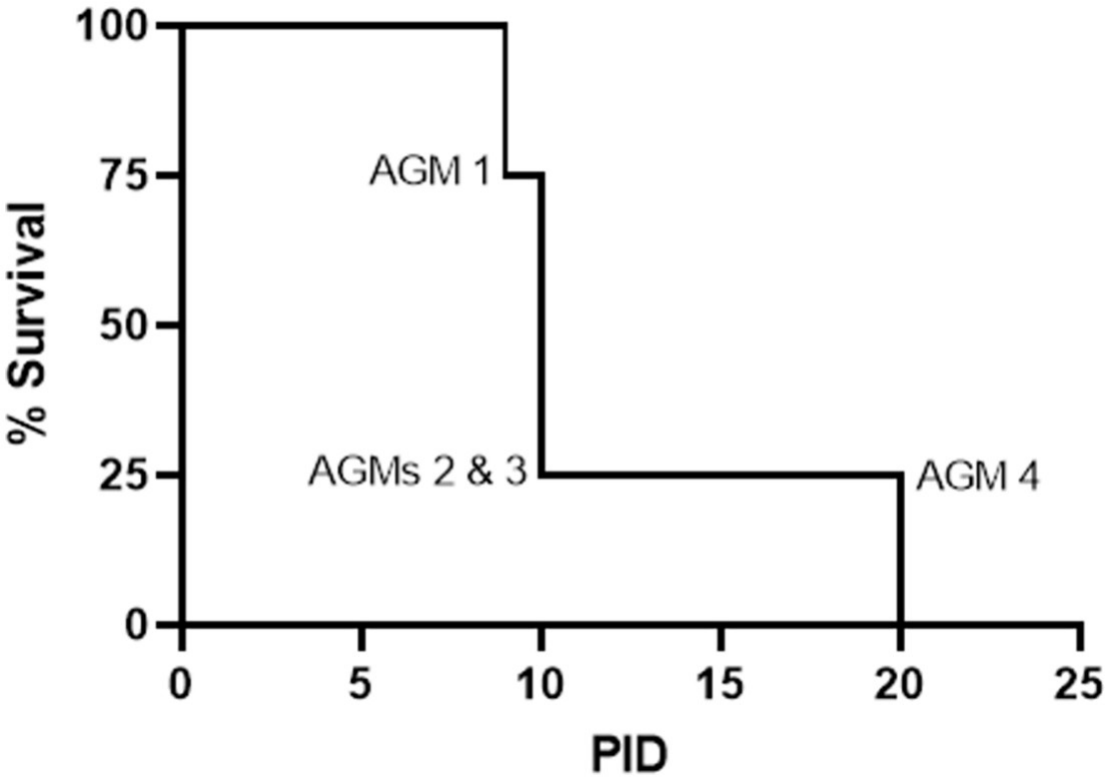
Kaplan-Meier survival curve. Percent survival is shown in this Kaplan-Meier survival curve.

**Fig 2:**
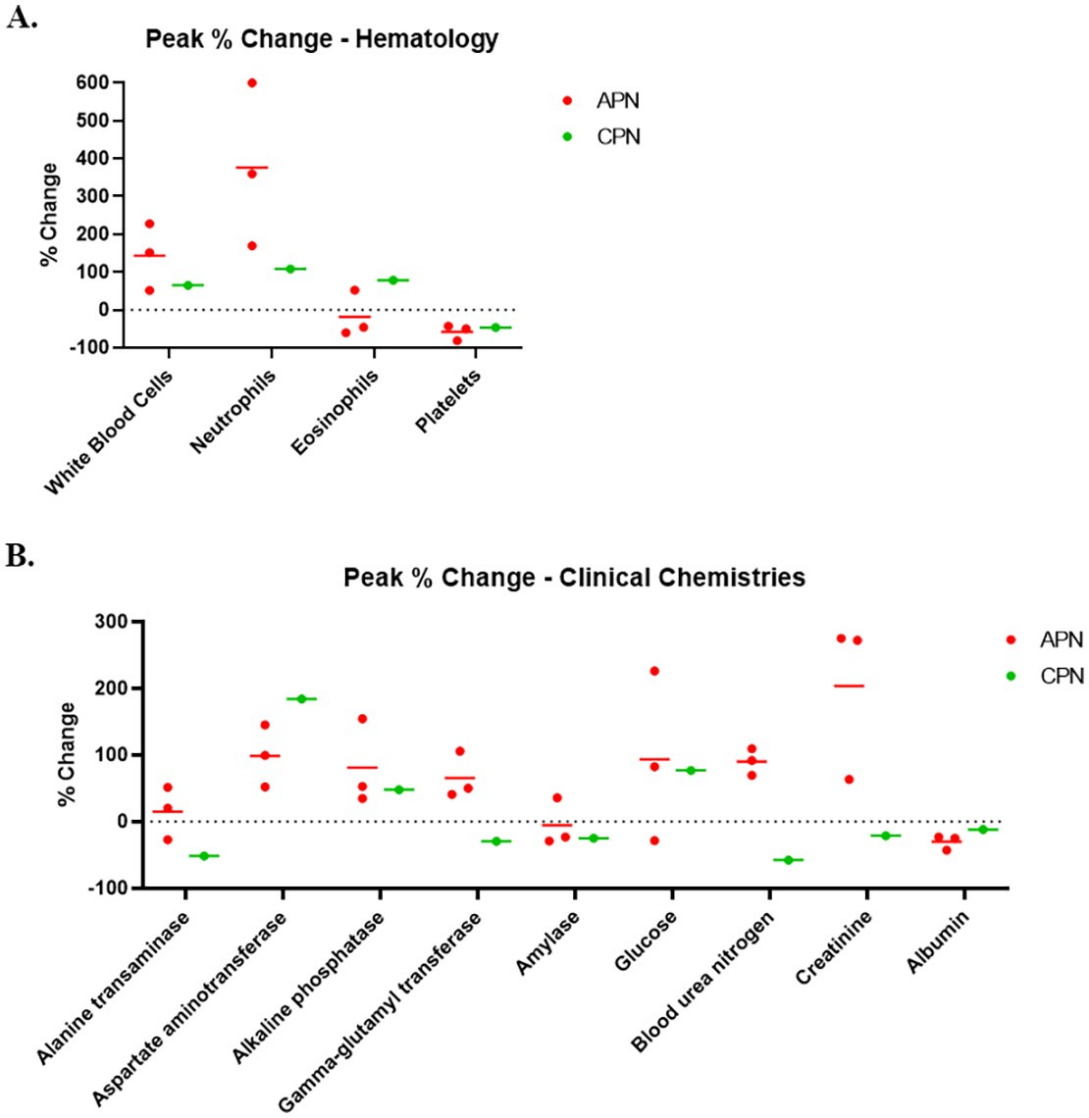
Percent change in hematology and clinical chemistry parameters. The percent change from baseline was determined for each animal for hematology and clinical chemistry parameters. Shown in this figure are peak percent change values for APN (red) and CPN (green) for relevant hematology (A) and clinical chemistry (B) parameters.

All animals succumbed or were euthanized as a result of NiV disease (Fig 1). AGMs 1 and 2 succumbed to disease and AGMs 3 and 4 were euthanized. All four animals developed clinical signs of disease characteristic of the acute phase, including decreased activity and responsiveness and labored breathing, with one animal (AGM 3) exhibiting signs of neurologic disease (i.e. a continuous head twitch noted during anesthetized observation on PID 10). Fever, defined as a greater than or equal to 1.5°C increase above baseline (average of rectal temperatures from PID -8, -1, and 0 for each animal), was measured on one or more PIDs for all but one animal (AGM 1), and a neutrophilic leukocytosis indicative of active infection was noted between PID 7–10 for all animals on study (Fig 2 and S1 Fig). AGMs 1–3 reached a terminal disease state during the previously-defined critical period [18] (PID 9 and 10). AGM 4 survived to PID 20. This animal developed acute disease similar to the animals that succumbed or were euthanized on PID 9–10. This animal remained clinically ill until PID 14 when he began to recover, and it was at this same time that eosinophil numbers were elevated (Fig 2 and S1 Fig). A new onset of clinical disease characterized by neurological deficits was noted by PID 17, and by PID 20 the animal was euthanized due to severe neurological disease.

In the following sections, a systematic approach will be taken to describe the pathological changes associated with disease in these animals. Where applicable, these changes will be discussed in relation to clinical disease findings obtained during cage side or anesthetized observations. As defined by Johnston *et al*., incubation is the period between PID 0–5, acute phase is the period between PID 5–12, the critical period is PID 9–12, and chronic phase is the period after PID 12 [18]. For simplicity, animals that succumbed/were euthanized during the critical period will be referred to as “acute phase NHP” or APN (AGM 1–3), and the animal euthanized during the chronic phase will be referred to as “chronic phase NHP” or CPN (AGM 4). Baseline for clinical pathology evaluations is defined as the mean of the values obtained from PID -8, -1, and 0 for a given analyte for each animal.

#### Respiratory system

**Fig 3:**
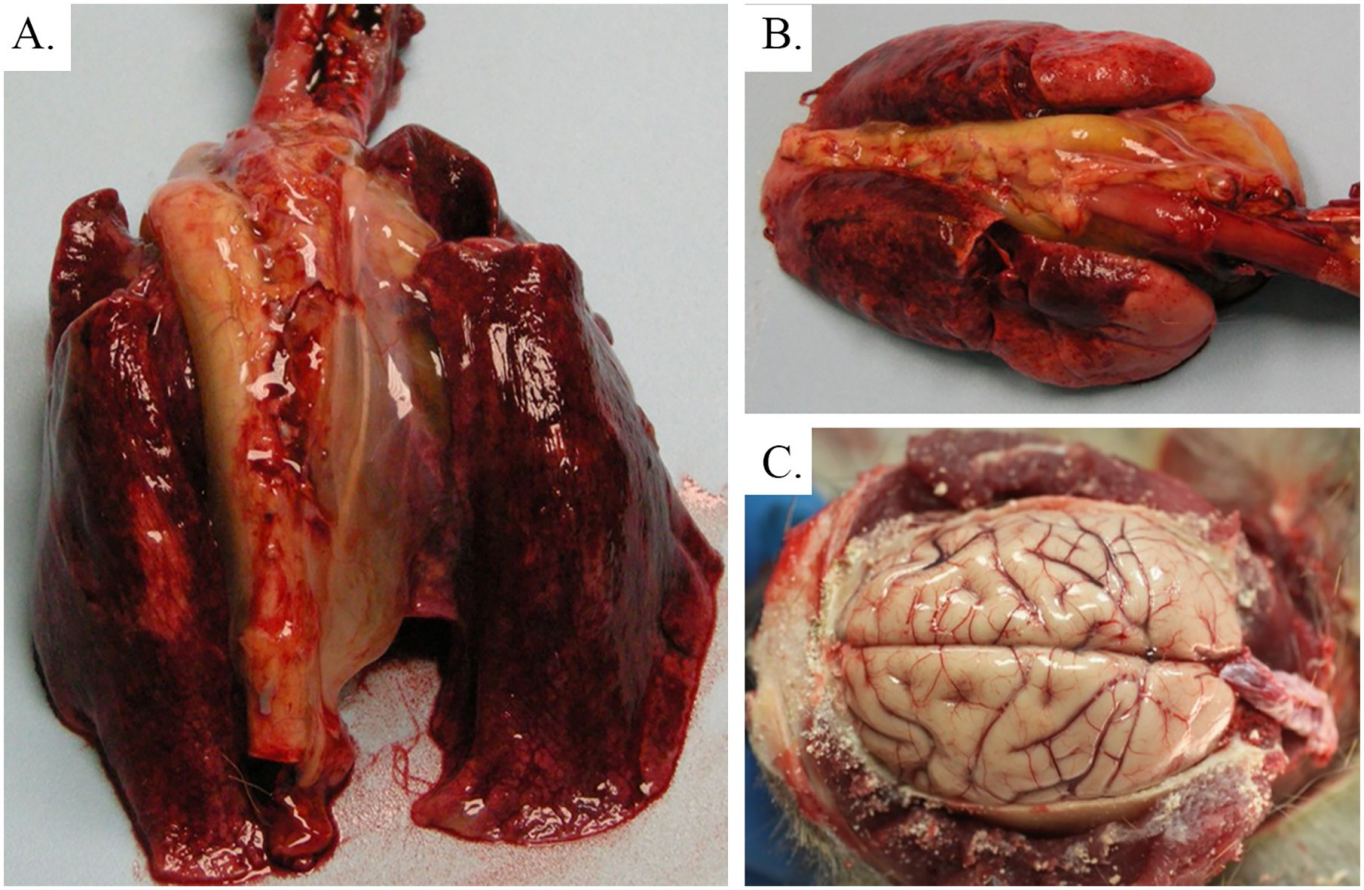
Lung and brain—Gross pathology images. Image A is a gross image of the lungs from AGM 1, showing mottled, congested, wet and heavy lungs that fail to completely collapse, with prominent mediastinal edema. Similarly, image B is a gross image of the lungs from AGM 3, showing mottled, congested, wet, heavy lungs that fail to completely collapse. Image C is the brain of AGM 4, demonstrating diffuse, moderate congestion of the meninges.

**Fig 4:**
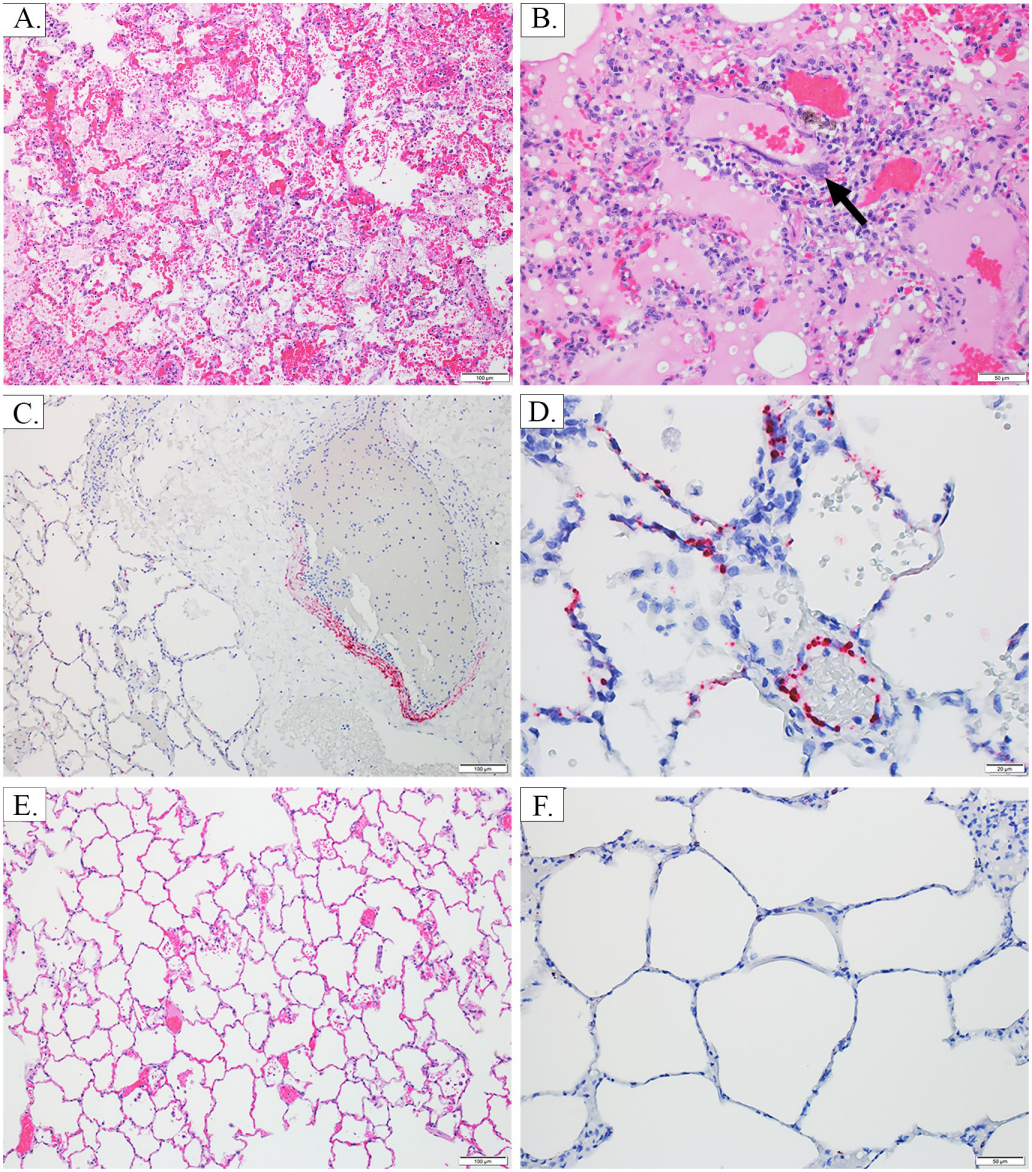
Lung—H&E and ISH. Image A, AGM 2, Lung 10X H&E: Alveolar septa are expanded by inflammation and congestion, and alveolar lumina contain moderate amounts of fibrin and hemorrhage. Image B, AGM 3, Lung 20X H&E: Alveolar septa are expanded by inflammation and edema which extends into and fills alveolar lumina, accompanied by small amounts of hemorrhage; rare viral syncytia are present within the tunica intima of vessels (black arrow). Image C, AGM 1, Lung 10X ISH: There is NiV labeling in the tunica media and intima of a pulmonary artery, as well as in the adjacent alveolar septa. Image D, AGM 3, Lung 40X ISH: There is NiV labeling in the tunica intima of a small vessel, as well as in the adjacent alveolar septa. Image E, AGM 4, Lung 10X H&E: Essentially normal lung, absence of inflammation in the CPN. Image F, AGM 4, Lung 20X ISH: Viral labeling is absent from alveolar septa, which are essentially normal in the CPN.

**Fig 5:**
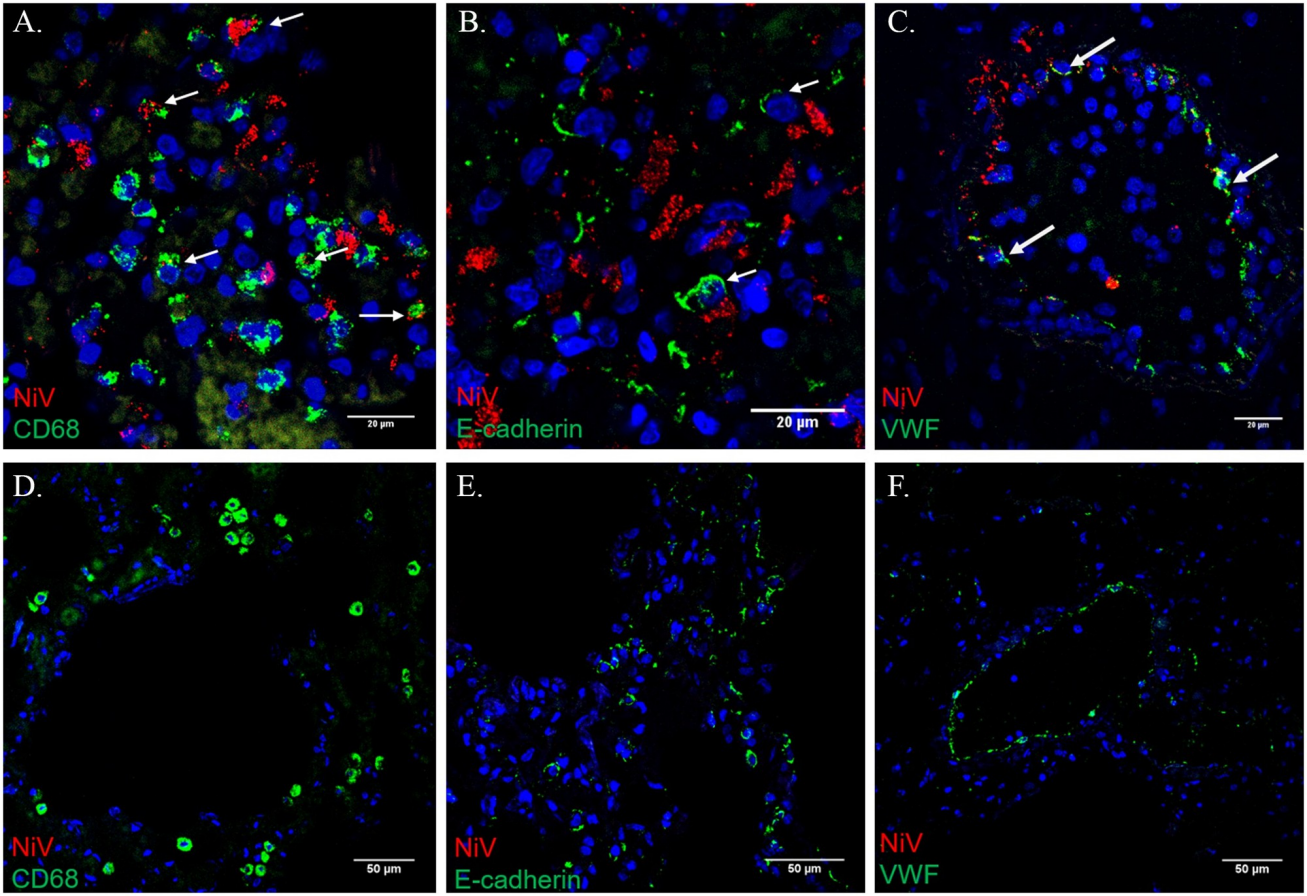
Lung—IFA. Image A, AGM 1: Immunofluorescence labeling demonstrates NiV antigen (red) detected in CD68+ macrophages (green), identified by white arrows. Image B, AGM 1: Immunofluorescence labeling demonstrates NiV antigen (red) is not detected in E-Cadherin+ pneumocytes (green), identified by white arrows. Image C, AGM 1: Immunofluorescence labeling demonstrates NiV antigen (red) is detected in Von Willebrands factor+ endothelial cells (green), identified by white arrows. Image D, AGM 4: NiV antigen (red) was not detected in CD68+ macrophages (green). Image E, AGM 4: NiV antigen (red) was not detected in E-cadherin+ pneumocytes (green). Image F, AGM 4: NiV antigen (red) was not detected in VWF+ endothelial cells (green).

**Fig 6:**
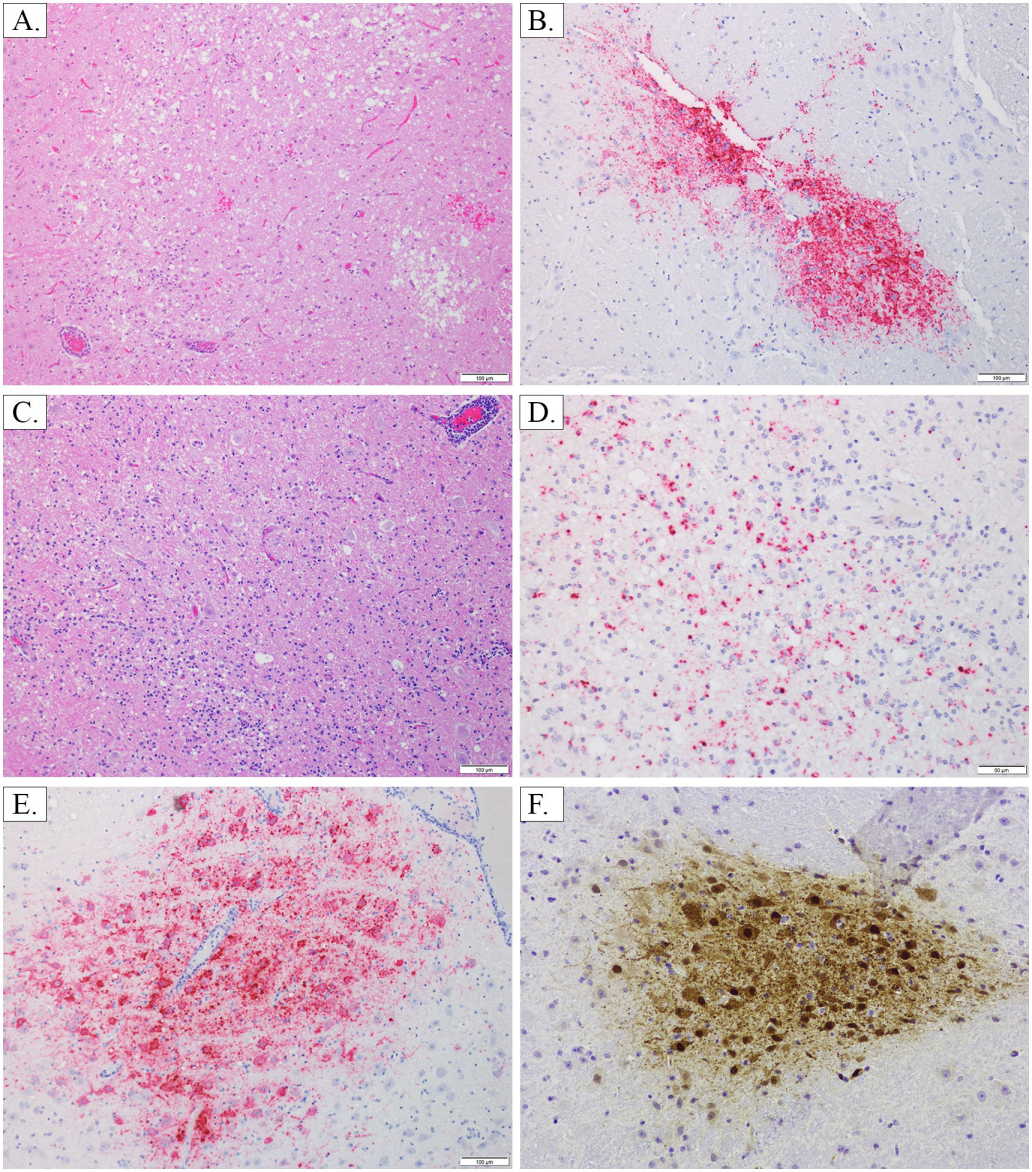
Brain- H&E, IHC, ISH. Image A, AGM 1, brain, pons 10X H&E: Focally extensive area of spongiosis and rarefaction of neuropil with gliosis, hemorrhage and perivascular cuffing. Image B, AGM 1, brain, pons 10X ISH: NiV labeling in the neuropil, glia and neuron cytoplasm in an area of neuropil rarefaction and gliosis (roughly corresponding to image A). Image C, AGM 4, brain, medulla 10X H&E: An area of moderate gliosis and inflammation with perivascular cuffing and spongiosis. Image D, AGM 4, brain, medulla 20X ISH: NiV labeling in an area of inflammation and gliosis (roughly corresponding to image C). Image E, AGM 4, brain, pons 10X ISH: NiV labeling in the neuropil, neuron cytoplasm and glia in an area without significant inflammation or gliosis. Image F, AGM 4, brainstem, 20X IHC: Positive NiV labeling in neurons and adjacent neuropil.

**Fig 7:**
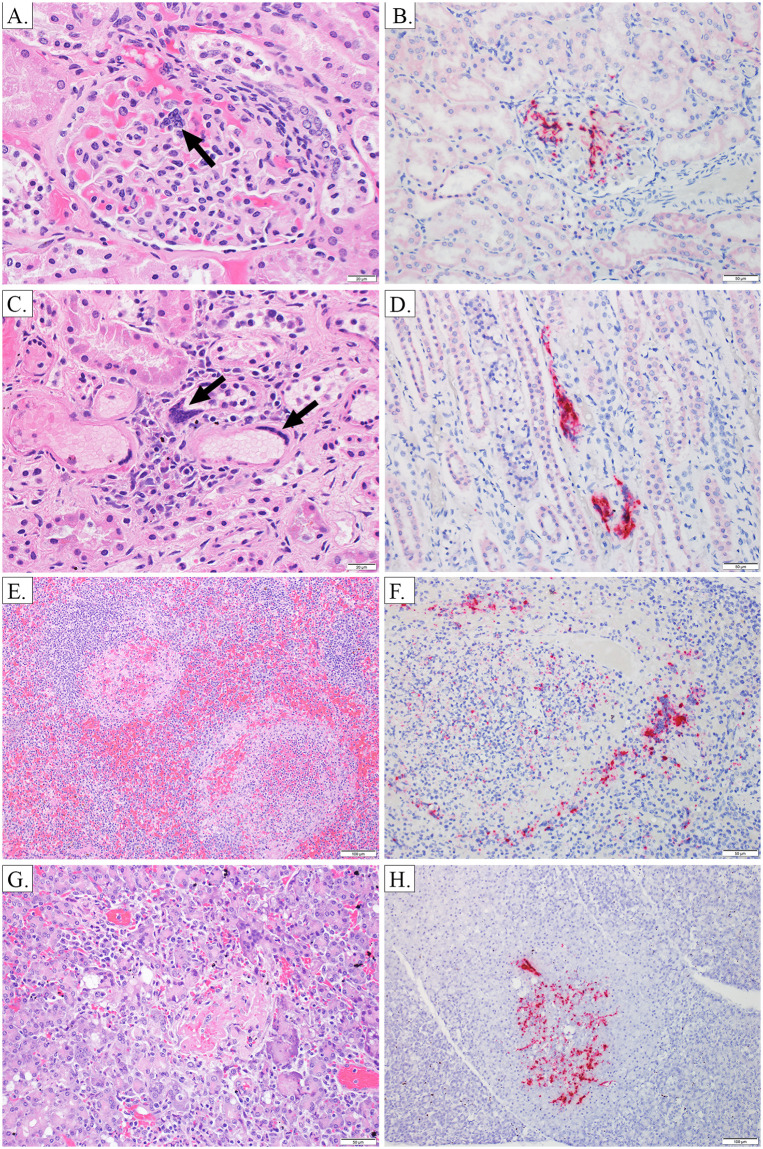
Kidney, spleen, pancreas—H&E and ISH. Image A, AGM 2, kidney 40X H&E: Viral syncytia in the renal glomerulus. Image B, AGM 1, kidney 20X ISH: NiV labeling in the renal glomerulus. Image C, AGM 2, kidney 40X H&E: Viral syncytia in vessels in the renal interstitium. Image D, AGM 2, kidney 20X ISH: NiV labeling in the renal interstitium. Image E, AGM 3, spleen 10X H&E: Multifocal areas of necrosis with abundant fibrin, hemorrhage and inflammation effacing the white pulp architecture. Image F, AGM 1, spleen 20X ISH: NiV labeling within and surrounding areas of necrosis, including in viral syncytia. Image G, AGM 1, pancreas 20X H&E: Focal area of pancreatic degeneration and necrosis with hemorrhage and fibrin. Image H, AGM 1, pancreas 10X ISH: Focal area of NiV labeling corresponding to an area of degeneration/necrosis.

The most prominent disease sign for APN was severe respiratory distress characterized by elevated respiratory rates and labored breathing, often necessitating euthanasia. Frequently, open mouth breathing and the use of accessory muscles to breath were noted, and crackling was felt in the chest of APN. Images related to this section can be seen in Figs 3–7, and a summary of histologic, IHC and ISH findings can be found in Tables 1–3. At necropsy, the lungs were red, mottled, and edematous (failed to collapse). Mediastinal edema was noted, along with pleural effusion and the presence of approximately 10 mL of blood-tinged serosanguinous fluid in the thoracic cavity. Histopathological evaluation revealed moderate to marked, multifocal interstitial and lymphohistiocytic pneumonia with vasculitis, fibrin, hemorrhage, and edema. Necrosis and the presence of a few syncytial cells (evidence of NiV-MY infection) were observed in lung sections from AGM 2–3. All APN had NiV antigen staining associated with endothelium and alveolar macrophages, and macrophage staining was multifocal. AGM 2–3 had very strong multifocal staining of endothelium (including of the smooth muscle of the tunica media) that was given an IHC score of 2–3+. AGM 1 also had very strong multifocal staining of the endothelium (including the smooth muscle of the tunica media) that was more widespread (IHC score of 3–4+) than that observed for AGM 2–3. NiV antigen was detected by IFA in CD68+ macrophages, but not in E-cadherin+ pneumocytes. Additionally, NiV antigen was also detected in vascular endothelial cells labelled by anti-VWF antibodies. Therefore, all APN had evidence of active NiV infection in the lung tissue. These findings are generally consistent with those previously reported for AGM [6, 18–21] and correlate with the severe respiratory distress observed clinically for APN’s.

**Table 1:** Summary of histologic findings.

Animal #	1	2	3	4
Day/death PI	10	9	10	20
Pneumonia, interstitial	x	x	x	
Systemic vasculitis	x	x	x	x
Splenitis, necrotizing	x	x	x	
Pancreatitis, necrotizing	x	x	x	
Adrenal gland—necrosis	x	x	x	
Gliosis +/- necrosis, rarefaction and spongiosis	x	x	x	
Encephalitis, mononuclear with vasculitis				x
Meningitis, mononuclear				x

**Table 2:** Immunohistochemistry findings.

	Tissue localization	NHP 1	NHP 2	NHP 3	NHP 4
Lung	Endothelium	3–4, +, MF	2–3, +, MF	2–3, +, MF	BK
	Vessel smooth muscle	3, +, MF	2, +, MF	2, +, MF	
	Alveolar macrophages	1	1, MF	1, MF	
Heart	Endocardium, epicardium, tunica media	1, MF	1, MF	1, MF	ND
	Mononuclear intravascular cells	1–2, MF	1–2, MF	1, MF	
Spleen	Area of necrosis	4, +, MF	N/A	N/A	BK
	White pulp	3, F	2, MF	3–4, +, F	
	Vessel smooth muscle	1–2, MF	1–2, MF	1–2, MF	
Kidney	Inflammation, vessels, epithelium	2, +, MF	1–2, +, MF	1–2, +, MF	ND
Adrenal gland	Cortex, endothelium	3–4, +, MF	1, MF	ND	ND
	Corticomedullary junction	ND	ND	3, MF	
Skeletal muscle	Vessel smooth muscle, endothelium	2, F	ND	ND	
Pancreas	Necrosis, acinar epithelium	4, +, MF	4, +, MF	2–3, MF	BK
Small intestine	Vessel smooth muscle	1, MF	BK	BK	ND
Cecum	Vessel smooth muscle	2, +, MF	BK	2+	
Large Intestine		ND	BK	BK	
Cerebrum	Endothelium, neurons, +/- extraneuronal	1–4, +, MF	1, MF	1, MF	BK
Brainstem	Neurons, +/- extraneuronal	4, +, MF	1, MF	1, MF	2–3, MF
	endothelial	1, MF	1, MF	1, MF	ND
Cerebellum	Neurons, +/- extraneuronal	4, +, MF	1, MF	4, +, F	2, MF
	endothelial	1, MF	1, MF	1, MF	ND
Lymph node	All	NP			
	Axillary		BK	3, +, MF	NP
	Mesenteric		NP	NP	ND
Eye		ND	ND	ND	NP

1 = 1–10 cells/high power field (hpf).

2 = 11–20 cells/hpf.

3 = 21–40 cells/hpf.

4 = >40 cells/hpf.

D = Diffuse.

F = Focal.

MF = Multifocal.

+ = intense staining.

ND = Not detected.

NP = Not performed

BK = Background staining inhibiting interpretation.

**Table 3:** *In situ* hybridization findings.

	Tissue localization	NHP 1	NHP 2	NHP 3	NHP 4
Lung	Alveolar septa (predominant); vascular smooth muscle; endothelium; exudate	3	2	2	ND
Spleen	Areas of necrosis; white and red pulp; syncytia	3	1	2	ND
Kidney	Vascular smooth muscle; interstitial and glomerular vessels;	2	1	1	ND
Adrenal gland	Cortex; medulla	1	ND	1	ND
Pancreas	Exocrine pancreas, necrosis, islets	2	1	1	ND
Duodenum	Lamina propria	1	1	1	ND
Mesentery	Vascular smooth muscle	1	ND	ND	ND
Cerebrum	Corpus striatum-neuronal; extraneuronal; vessels; meningeal vessels	1	ND	1	ND
Brainstem, pons	Area of necrosis—neuronal, extraneuronal; meninges	2	ND	ND	
	Neuronal, extraneuronal (no necrosis present)				1
Brainstem, medulla	Neuronal, extraneuronal	1	ND	ND	
	Neuronal, extraneuronal, area of inflammation/gliosis				1
Cerebellum	Meningeal vessels (smooth muscle, endothelium)	1	ND	ND	ND
TB Lymph node	Endothelium, other	1	ND	NP	NP
Eye		NP	NP	NP	ND

Intensity of staining was graded on the following scale:

Minimal = If 10% or less of the cells in the section are immunoreactive, then score is 1.

Mild = If between 11% and 25% of the cells in the section are immunoreactive, then score is 2.

Moderate = If between 26% and 50% of the cells in the section are immunoreactive, then score is 3.

Marked = If between 51% and 79 of the cells in the section are immunoreactive, then the score is 4.

Severe = If between 80% or more the cells in the section are immunoreactive, then the score is 5.

ND = Not detected

NP = Not performed.

The CPN also exhibited signs of respiratory disease including an elevation in respiratory rate and the presence of crackling in the chest; however, these signs were much less severe than those noted for APN and resolved by PID 14. At necropsy, respiratory system tissues (including the lungs) were grossly within normal limits, and no lesions were noted upon histologic examination. There was artifactual background labeling in the lung of the CPN with the IHC procedure that interfered with interpretation, but viral labeling by ISH was absent in the lung of this animal (see images E and F of Fig 4 and Tables 1–3). Additionally, NiV antigen was undetectable in CPN using IFA in macrophages, pneumocytes and endothelium (see Fig 5, images D-F).

#### Nervous system

Neurological signs of disease were largely absent for APN with the exception of AGM 3 which had a continuous head twitch noted during terminal anesthetized observation on PID 10. Images related to this section can be seen in Fig 6, and a summary of histologic, IHC and ISH findings can be found in Tables 1–3. AGM 1 and 2 had moderately congested meninges at necropsy. Histopathologic examination revealed minimal to moderate multifocal gliosis (± rarefaction and spongiosis) in the cerebrum, cerebellum, brainstem, and pons for all APN. Both AGM 2 and AGM 3 had multifocal staining of endothelium of the cerebrum, cerebellum, and brainstem that was given an IHC score of 1, and both animals had staining of neurons in the cerebrum, cerebellum, and brainstem that was multifocal and given an IHC score of 1 with the exception of staining in neurons of the cerebellum for AGM 3 that had focal, very strong, and widespread (IHC score of 4+) staining. For AGM 1, multifocal NiV antigen staining associated with endothelial cells and, rarely, neurons in the frontal cortex and corpus striatum of the cerebrum was given an IHC score of 1. Neuronal and extraneuronal staining of the thalamus (cerebrum), pons, and cerebellum was very strong, focal, and widespread (IHC score of 4+) for AGM 1.

Minimal (score of 1) viral labeling by ISH was present in the cerebrum, cerebellum and medulla of AGM 1; mild viral labeling (score of 2) was present in the pons. There was an absence of ISH labeling in the brain of AGM 2, and AGM 3 only had a small amount of ISH viral labeling in the cerebrum. ISH labeling, when present, was both neuronal and extraneuronal and in some cases was identified in vessels, the meninges, and areas of necrosis and inflammation (see Table 3 and Fig 6 images A and B). These APN had markedly depressed activity due to severe respiratory compromise which may have masked the ability to recognize and evaluate clinical signs associated with neurologic disease.

Based upon clinical observations, the CPN (AGM 4) had recovered from acute phase disease by PID 14. During the PID 17 observation, this animal was found drooling and stumbling and by PID 18, the animal appeared very weak and his mouth was hanging open. Continuous tremors in the legs were observed on PID 19, and by PID 20 the animal was unable to stand or climb and was euthanized due to severe neurological deficits. During the terminal anesthetized observation, tremors and decreased muscle mass in the legs were noted. At necropsy, the superficial cerebral vessels were moderately congested (see Fig 3, image C). Histologically, moderate to marked multifocal encephalitis in the brainstem and cerebellum, with lymphohistiocytic vasculitis, gliosis, rarefaction, spongiosis, and meningitis were noted. Consistent with prior studies [18], the neurons and extracellular spaces within the brainstem and cerebellum stained multifocally for NiV antigen (IHC score = 2–3), with some staining observed in macrophages or dendritic cells based on their histomorphology. A minimal degree (score of 1) of neuronal and extraneuronal ISH labeling was present in areas of inflammation in the brainstem and areas of gliosis in the medulla (see Table 3 and images C, D, E and F of Fig 6 for details). Collectively, these findings are suggestive of an active CNS infection, which correlates with the clinical findings on PID 20. This animal developed neurologic disease during the chronic phase similar to that reported elsewhere for the AGM model of NiV-MY i.t. infection [17, 18].

The sciatic nerve, brachial plexus, and eyes were also examined in this study. In all cases (APN and CPN), no significant findings were associated with these tissues.

#### Digestive system

The tissues of the digestive system examined during this study included the pancreas, liver, small intestine, cecum, colon, stomach, gallbladder, and esophagus. Representative histological and ISH images of the pancreas can be found in Fig 7 images G and H, with findings related to digestive system tissues also summarized in Tables 1–3. Necrotizing pancreatitis with the presence of syncytial cells was present in the three APNs. Labeling for NiV antigen was present in areas of pancreatic necrosis in the APN, with a score ranging from 2–4 by IHC and minimal to mild (score 1–2) by ISH. Aside from the pancreas, IHC antigen staining when observed was associated with blood vessels and was likely the result of infection of various cell types within the vessel wall. Viral ISH labeling was observed in the lamina propria of the duodenum in all APN. Clinical pathologic changes observed for APN, including reductions in amylase levels and elevated serum glucose levels, were consistent with the damage observed in the pancreas histologically (Fig 2 and S3 Fig).

No noteworthy histopathologic lesions were noted in the liver in the APN or CPN. The following enzymes indicative of liver function were assessed on this study: alanine transaminase (ALT), aspartate aminotransferase (AST), alkaline phosphatase (ALP), and gamma-glutamyl transferase (GGT). AST and ALP were elevated for APN and CPN, and GGT was elevated for APN (Fig 2 and S2 Fig). Although these three enzymes are typically indicative of damage to the liver, an absence of liver pathology for NiV-infected AGM suggests another etiology. Elevations of AST can also indicate damage to or inflammation of the pancreas, which was observed pathologically on this study. ALP and GGT elevations can also indicate cholestasis due to various causes including bile duct obstruction; this cannot be ruled out as a cause for the elevation of these enzymes on this study, but evidence for such was not observed histologically. ALP elevations may also indicate damage/inflammation of the gallbladder; however, this tissue was histologically normal in APN and CPN, suggesting this was not the main cause of the elevated levels of this enzyme. Collectively, histopathological and clinical chemical data indicate that the liver is likely only minimally affected during NiV-MY infection in AGM. However, passive hepatic congestion potentially associated with hypoxia related to increased lung pathology has been reported in NiV-BD infection in the AGM [7].

Gross, histopathological findings associated with damage resulting from NiV-MY infection were absent in the pancreas and liver of the CPN. In addition, viral antigen staining was absent from these tissues for this animal, and viral nucleic acid was not detected by ISH. However, evidence of likely prior pancreatic damage/inflammation was present, as amylase levels were reduced by PID 10, and serum glucose levels were elevated at the terminal time point (PID 20).

Significant histologic lesions were not observed in the small intestine, cecum, colon, stomach, gallbladder or esophagus for APN or CPN on this study.

#### Endocrine system

Similar to what was found in the digestive tract, the major findings in the endocrine system (summarized in Tables 1–3) included necrotizing vasculitis that was found in the thyroid gland of 2/3 APN’s (AGM 2 and AGM 3). Histologic findings included moderate to marked multifocal lymphohistiocytic vasculitis, fibrin, hemorrhage, edema, single cell necrosis, with scattered syncytial cells. Minimal to mild multifocal necrosis was present in the cortex of the adrenal gland of all three APN’s.

Viral antigen IHC labeling in the adrenal gland ranged from a score of 1–4 in the APN and included labeling in the cortex, endothelium and corticomedullary junction (see Table 2). A minimal (score of 1) amount of viral ISH labeling was observed in the cortex and medulla for AGMs 1 and 3. These findings are consistent with results from prior studies [18, 25] and suggest that the adrenal gland may be a specific target for NiV-MY in acutely infected AGM.

No significant findings were noted in the adrenal gland or thyroid gland for the CPN.

#### Lymphatic system / immune system

Lymphatic / immune tissues examined on this study included the following: lymph nodes, spleen, and thymus. Representative histological and ISH images can be found in Fig 7, images E and F, with findings related to these tissues also summarized in Tables 1–3. For all APN, the spleen was mildly enlarged at necropsy and oozed blood on the cut surface. Histopathologically, multifocal necrotizing splenitis was present in both the red and white pulp, characterized by the presence of necrotic debris along with fibrin, hemorrhage, edema and scattered syncytial cells. Necrosis was often most prominent in the white pulp, and there was also an overall decrease in the density of white pulp. Splenitis was moderate for AGM 1, mild for AGM 2, and moderate to marked and suppurative with vasculitis for AGM 3. The spleen of AGM 2 had a different appearance compared to APN’s 1 and 3 with the necrosis being more diffuse and with some loss of overall tissue architecture, more suggestive of coagulative necrosis, as may be seen in cases of infarction. For all 3 APN, NiV antigen staining was associated with vessels (smooth muscle of the tunica media, multifocal, IHC score of 1–2) and the white pulp; additional staining for AGM 1 was noted in areas of necrosis (focal and very strong, with an IHC score of 4+). Antigen staining intensity and distribution in the white pulp was varied for the 3 APN. For AGM 1, staining was focal with an IHC score of 3. For AGM 2, staining was multifocal with an IHC score of 2. For AGM 3, staining was multifocal and very strong, with an IHC score of 3–4+. Viral ISH labeling was present in the spleen of all three APN and ranged from minimal (score of 1) to moderate (score of 3). Labeling was present in areas of necrosis, in both the white and red pulp as well as in syncytia (see Table 3 for details). These data indicate that the spleen was actively infected with NiV in APN and, thus, represents a prominent target tissue for NiV during infection in AGM. These histopathology findings for the spleen are generally similar to those reported previously, which have included degeneration or necrosis in the white pulp, variable vasculitis and fibrin deposition, and the presence of syncytia and viral antigen in histiocytes, syncytia and/or endothelial cells [6, 18–21].

For the CPN, the spleen was mildly enlarged at necropsy, with prominent white pulp on the cut surface. Rare syncytial cells were observed histologically in the spleen. Rare syncytial cells, although not necessarily indicative of active NiV infection, are a hallmark of either previous or active NiV infection, and their presence indicates that the spleen was a target for NiV infection in the CPN. Viral ISH labeling was not observed in the spleen of the CPN.

Outside of lymphoid depletion and rare syncytial cells, no significant findings were associated with the thymus for any animals on this study.

The mediastinal, mesenteric, tracheobronchial (APN only), inguinal (APN only), submandibular (AGM 1 only), and axillary (AGM 2–4 only) lymph nodes were examined on this study. Similar to prior studies [18, 19], the predominant finding in lymph nodes of APN consisted of mild to moderate diffuse lymphoid depletion of germinal centers with lymphocytolysis, fibrin, hemorrhage, edema, vasculitis, and sinus histiocytosis. For the CPN, mild diffuse follicular hyperplasia with sinus histiocytosis was observed in the mediastinal, mesenteric, and axillary lymph nodes, and there was an absence of IHC staining for NiV antigen. Abundant (3+ score) IHC staining for NiV antigen was present in the axillary lymph node in AGM 3; IHC was either not performed in lymph nodes of the other animals or abundant background labeling inhibited accurate interpretation of results.

#### Urinary system

Tissues of the urinary system examined on this study included the kidney and urinary bladder. Representative histological and ISH images of the kidney can be found in Fig 7, images A-D, with findings related to these tissues also summarized in Tables 1–3. Grossly, the urinary bladder was diffusely congested in AGM 1, and there were petechial hemorrhages on the mucosa in AGM 3. These findings are similar to those previously reported for this tissue [18–21, 25]. Histological findings associated with the urinary bladder in AGM’s 2 and 3 consisted of lymphohistiocytic cystitis with vasculitis that varied from minimal to moderate depending on the APN examined. No significant findings were noted in the urinary bladder of AGM 1 or the CPN. Viral antigen staining was not performed on the urinary bladder on this study.

As previously documented [18, 19], the kidney appeared to be a prominent target tissue for NiV. For APN, mild multifocal lymphohistiocytic interstitial inflammation with endothelial and tubular epithelial syncytial cells and rare necrotic tubular epithelial cells were noted. In addition, vasculitis within the kidney was noted for AGM 3, and tubular ectasia was noted for AGM 1. These findings were associated with active NiV infection as areas of inflammation (mononuclear cells), endothelium, the tunica media, and epithelium were very strongly positive multifocally for NiV antigen (IHC score of 1–2+). Viral ISH labeling ranged from a severity score of 1–2 (minimal to mild) in the three APNs, and included labeling in vascular smooth muscle in larger vessels as well as smaller vessels in the interstitium and glomeruli.

Azotemia as a marker of NiV disease in AGM has been previously reported [18]. In the present study, azotemia, as evidenced by elevated levels of blood urea nitrogen and creatinine, was detected at terminal time points for all APN (Fig 2 and S3 Fig). Based on the level of kidney damage observed histologically, it could be predicted that this finding represents renal azotemia (as opposed to pre-renal or post-renal azotemia); however, without urine specific gravity assessments (which were not performed on this study), this cannot be conclusively determined.

Similar to APN, the CPN had mild multifocal lymphohistiocytic interstitial inflammation with vasculitis in the kidney. Syncytia were not observed, and no significant antigen staining was noted in the CPN. Viral ISH labeling was also not detected in the kidney of the CPN. These findings suggest active NiV infection in the kidney was absent at this later time point and the histological findings likely represent resolving lesions in the kidney from prior NiV infection.

#### Reproductive system

The tissues of the reproductive system examined on this study included the testes, prostate gland, and seminal vesicle. There were no significant findings in any of these examined tissues. Therefore, reproductive tissue does not appear to be a target for NiV in male AGM. Note, however, that this study did not contain any female AGM, so NiV infection of female reproductive tissues was not evaluated. Viral antigen or nucleic acid labeling was not performed on reproductive tissue.

#### Integument/musculoskeletal

No significant findings were associated with the haired skin of any APN or the CPN on this study. Outside of vessels within skeletal muscle which had IHC staining in AGM 1, no other significant findings were noted.

#### Cardiovascular system

Tissues of the cardiovascular system examined as part of this study included the heart, aorta, and blood vessels. Extensive systemic vascular damage associated with NiV infection has already been discussed in previous sections and, therefore, will not be described in full again here. However, it should be noted that the degree of damage for APN and the CPN is consistent with a vascular change that could be responsible for blood volume loss through hemorrhage and/or pleural effusion. Platelet reductions for APN and CPN (Fig 2 and S1 Fig), consistent with those described in Johnston *et al*. [18], likely occurred to some degree as a consequence of viral infection, but may have also been a component of disseminated intravascular coagulation; however, without data on other coagulation parameters this cannot be conclusively confirmed.

No significant findings were noted in the aorta for APN or the CPN. Histopathologic lesions were absent in the heart of the three APNs; however, minimal lymphohistiocytic vasculitis was present in the CPN. Conversely, immunohistochemical staining for NiV antigen was present in the tunica media of vessels in the heart, endocardium and epicardium, with an IHC staining intensity of 1 in the three APNs; staining was absent in the heart of the CPN. Infection of smooth muscle of the tunica media in vessels was noted for numerous tissues for APN including the lung (3/3, discussed in the pulmonary section), spleen (3/3, discussed in the lymphatic/immune system section), kidney (3/3, discussed in the urinary section), skeletal muscle (1/3, discussed above), small intestine (1/3, discussed in the digestive section), and cecum (2/3, discussed in the digestive section). In many cases, this finding correlated with vascular damage noted systemically in these animals, and is in agreement with previous studies which have demonstrated systemic vasculitis is a hallmark of NiV disease in both humans and AGM [15, 20, 25].

For the CPN, there were numerous vessels within the kidney (discussed in the urinary section) and heart (discussed above) that displayed signs of chronic vasculitis. These lesions were most likely resolving lesions from NiV infection. Viral antigen was not present within the inflammatory cell population or endothelial cells of the affected vessels, but the distribution and histologic changes suggest NiV infection as the cause. This pattern of infection and subsequent lesions in the APN and CPN may indicate early, minimal or mild infection of cardiac tissues (as seen in the APN), followed by prompt resolution with minimal observable residual histopathologic changes (as seen in the CPN).

**Fig 8:**
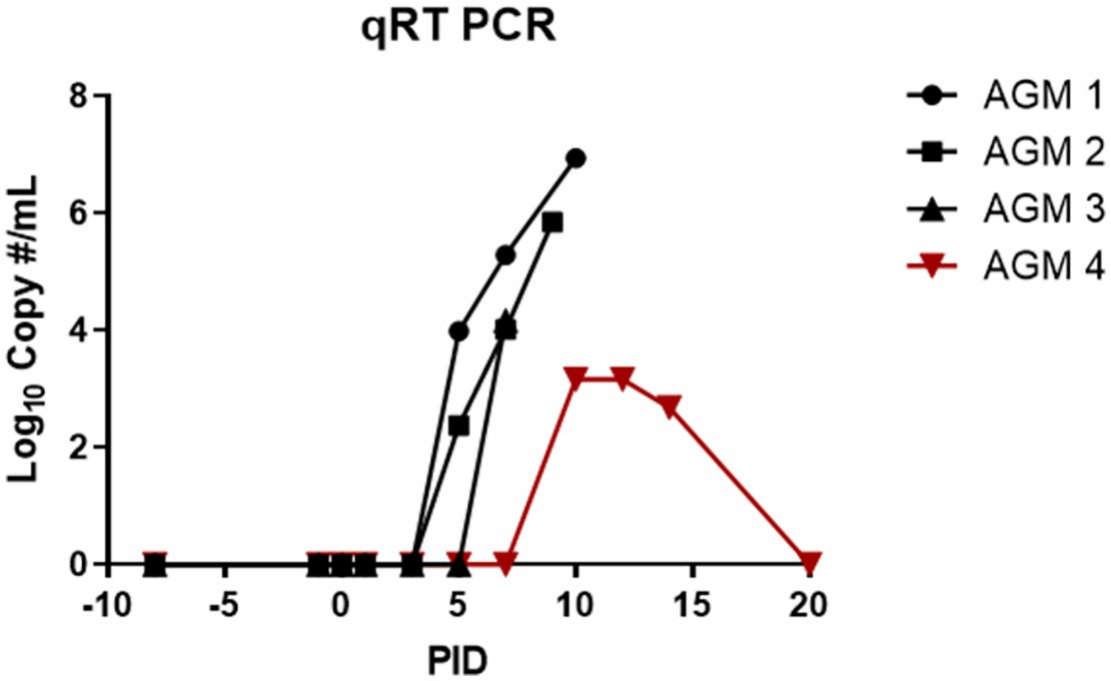
PBMC-associated viral RNA as detected by qRT-PCR. RT-PCR was performed on PBMCs for all animals on study. Shown is the Log_10_ copy #/mL for each animal. Black symbols are APN and the red symbols are the CPN.

**Fig 9:**
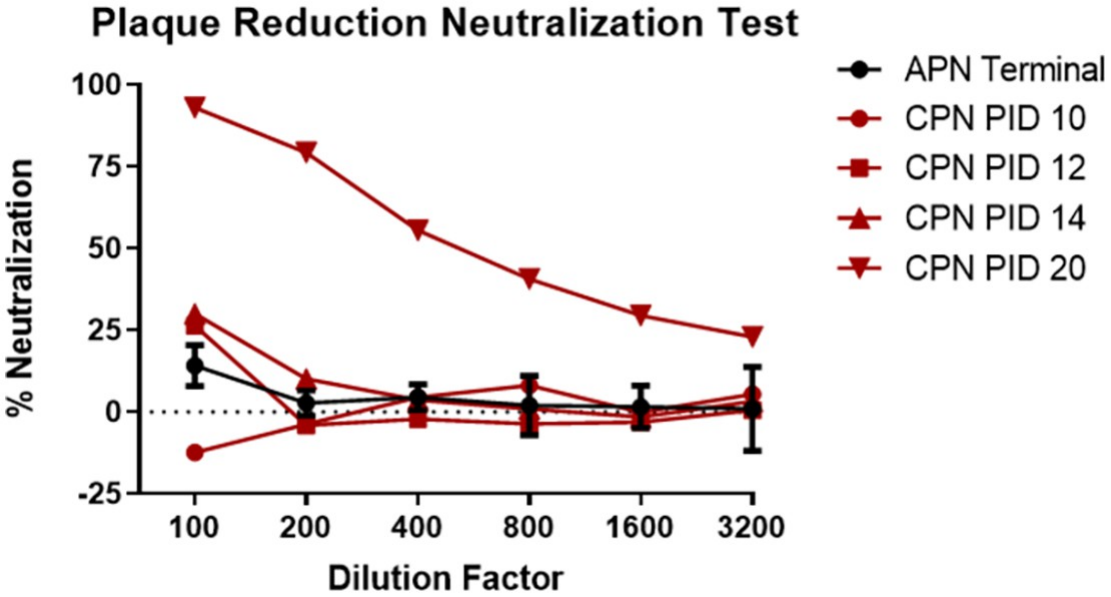
Neutralizing antibody responses as measured using a pseudovirion plaque reduction neutralization test. Shown in this figure is the percent of neutralization at various serum dilutions. Average terminal data for APN are show in black, with data for various PIDs shown in red for the CPN.

Consistent with data described in Johnston *et al*. [18], qRT-PCR performed on PBMCs (Fig 8) demonstrated the presence of circulating viral RNA in the blood by PID 5 (or 7 for AGM 3) for APN, with peak levels of 4.17–6.94 Log_10_ copies/mL at or near the time of terminal blood collection. Taken together with histologic and immunohistochemical findings in the blood vessels, these data are confirmatory of an ongoing systemic infection with NiV at the time of death. At the time that these animals succumbed or were euthanized, a neutralizing antibody response was not yet detectable (Fig 9).

PBMC-associated viral RNA for the CPN was not detected until PID 10, 3–5 days later than viral RNA was detected in the blood for APN. Peak levels of 3.16 Log_10_ copies/mL were noted on PID 10 and 12, and these levels were significantly lower than measured for APN. On PID 20, when this animal was euthanized due to severe neurological disease, NiV RNA was not detected in the blood. However, unlike APN, a strong neutralizing antibody response was noted for this animal. Neutralizing antibodies were first detected on PID 12 (26.41% neutralization at 1:100 dilution), with greater than 90% neutralization at a 1:100 dilution measured at terminal blood collection on PID 20. These data are similar to those presented in Johnston *et al*. [18] and suggest that a strong neutralizing antibody response develops after PID 12 during the chronic disease phase. Taken together with the histological findings for this animal, these data also demonstrate that systemic disease and viremia were largely resolved at the time of euthanasia for this animal even though neurological disease due to active viral infection in the brain was still ongoing.

## Discussion

The goal of the present study was to further characterize pathologic changes associated with the NiV-MY AGM i.t. model, and define how these changes relate to acute and chronic clinical disease as defined by Johnston *et al*. [18]. Four adult AGM were exposed to 2.41×10^5^ pfu of NiV-MY by the i.t. route using a bronchoscope MicroSprayer^®^ assembly, allowing the real-time visualization of viral deposition within the bilateral mainstem bronchi during administration. All 4 animals showed clinical signs of respiratory disease during the acute disease phase, and had histopathologic evidence of CNS infection; these findings are consistent with non-surviving animals described previously [18]. Three of four animals succumbed during the acute phase of the disease, and clinical findings for these animals were predominantly respiratory. The fourth animal was euthanized during the previously defined chronic phase [18] due to advanced stage neurologic disease.

The disease course defined for AGM in this study is similar to that described previously for respiratory routes of exposure [6, 17–19, 21], and similar temporal variation in clinical disease and survival has also been reported for humans [10, 15]. In AGMs exposed i.t. to NiV-MY, disease begins as a respiratory infection and the virus infects airway epithelium, endothelial cells, vascular smooth muscle, and alveolar macrophages (CD68+ monocytes/macrophages) resulting in interstitial pneumonia. The virus then spreads outside of the lungs, either cell free or attached to the cell surface [1, 26, 27], and infects vascular endothelial cells in organ systems throughout the body, smooth muscle cells of vessels, and scattered epithelial cells within the pancreas and adrenal glands. The precise time point (prior to day 9 post exposure) and route by which virus enters the brain during the course of infection is unclear, but several routes are possible and have been proposed. Entry could be hematogenous thru direct infection of endothelium, or by inflammatory cell trafficking generally through the vasculature of the CNS and/or specifically in the choroid plexus [1]. Entry into the CNS may also be facilitated or enhanced as a consequence of inflammatory signals within the blood-brain barrier (i.e. TNF-α), as well as of infection of specific leukocyte populations [28, 29]. Damage to the CNS may also occur primarily as a result of vascular damage, thrombosis and infarction resulting in CNS lesions consistent with ischemia [19]. In addition to a hematogenous route of infection, the olfactory route of infection into the CNS (i.e. olfactory epithelium, to olfactory nerve and into the olfactory bulb) has also been suggested and may occur in this animal model, although further investigation is necessary [1, 29, 30]. Munster *et al*. demonstrated that NiV entry into the brains of hamsters happens very early after intranasal exposure, occurring concurrent with infection of the lung tissue and prior to systemic spread [31]. If animals survive acute infection, viral clearance occurs in all organ systems with persistence in the central nervous system. It is presumed the virus may go latent, residing in neurons until reactivation and subsequent replication in the brain, which may lead to neurologic recrudescence that is reminiscent of relapse or late onset encephalitis observed in humans [12].

The pattern of disease noted for AGM on the present study, specifically the histopathologic lesions, closely mimics that seen in humans. Pulmonary lesions in patients infected with NiV include necrotizing alveolitis with the presence of multinucleated cells in the alveolar septum and lumen, with infection in endothelium and small vessel vasculitis; work in animal models indicates the early virus targets include bronchial epithelium and type II pneumocytes [15, 29, 32]. IFA results from the present study did not show infection of pneumocytes as was mentioned in a previous NiV AGM study [19]. We postulate, however, that infection of pneumocytes likely occurs very early in the course of infection, with clearance from pneumocytes by the end of the acute phase; at that time, NiV is predominantly located in macrophages, endothelium and other vascular cells. Lesions in the spleen in humans have been reported as white pulp depletion and necrotizing inflammation [15]. Lesions in the kidneys of humans include glomerular fibrinoid necrosis, vasculitis, thrombosis and interstitial inflammation as well as rare syncytia in glomeruli or tubular epithelium, similar to what has been reported here and elsewhere in the AGM model [15, 18]. Vasculitis has also been reported in small mesenteric arteries, the adrenal gland and pancreas in humans [15]. Primary lesions reported in the CNS include vasculitis, thrombosis and necrosis with varying degrees of inflammation and frequent viral inclusions [15]. Lesions present in cases of relapse encephalitis are isolated to the CNS, absent vascular lesions, and are characterized primarily by necrosis, edema and inflammation with the presence of viral RNA in neurons, glia and inflammatory cells [33].

The histopathologic CNS findings in this AGM modeling study included encephalitis, vasculitis, gliosis, rarefaction, spongiosis and meningitis, as well as labeling of virus in endothelium and neurons with a spatial variation in strength and location of labeling. These lesions resemble those seen in humans [15, 33]. Lee *et al*. described encephalitis with perivascular infiltrates and areas of encephalomalacia, perivascular cuffing and vasculitis following large-particle aerosol exposure of AGM to NiV-MY [17]. The areas of encephalomalacia were attributed at least in part to areas of ischemia that may be related to vasculitis.

The findings presented in Johnston *et al*., Lee *et al*., and in the present study indicate that a chronic phase neurologic disease is achievable in AGM following exposure to NiV-MY [17, 18]. Through the use of magnetic resonance imaging, Lee *et al*. was able to determine that lesions in the brain indicative of encephalitis likely occurred after PID 12. In addition, Liu *et al*. demonstrated that persistent/convalescent NiV-MY infection in the brain of AGM is possible, with labeling identifiable in neurons and microglial cells of the brainstem, cerebral cortex, and cerebellum at day 32 post exposure [34]. Johnston *et al*. also showed that significant neurological disease and prominent antigen staining were present in the brains of survivors at PID 32, including one survivor that appeared clinically healthy for the entire duration of the study [18]. Taken together, these findings indicate that chronic phase neurologic disease in the AGM is likely due to viral persistence in regions of the brain, resulting in encephalitis. These features of disease in the AGM model (i.e. survivors and viral persistence) may facilitate the study of “late onset” or “relapse” encephalitis described in humans. Relapse/late onset encephalitis can occur in up to 7.5% of humans that have recovered from acute phase disease following exposure to NiV-MY, and up to 3.4% of patients who were initially asymptomatic following an exposure to NiV-MY [12]. “Late onset” encephalitis associated with clinical NiV infection in humans has been defined in the literature as primary onset of encephalitis 10 or more weeks after virus exposure, and”relapse” has been defined as occurrence of neurologic symptoms after recovery from NiV encephalitis [11, 12, 14]. In the early NiV outbreaks in Malaysia, time from infection to onset of symptoms ranged from four days to two months, with the majority becoming symptomatic within two weeks of infection [10, 14], and death occurring within 1–2 weeks following initial symptom onset [35]. Johnston *et al*. defined the AGM incubation following i.t. exposure to NiV-MY as up to 5 days, with death from acute phase disease occurring during the critical period 9–12 days post-exposure [18, 25]. Based on data presented herein and by Lee *et al*. [17], death from neurologic disease may also occur during the previously-defined chronic phase (PID 12+) [18]. However, it is still difficult to conclusively call this “late onset” versus “relapse” encephalitis. Temporal monitoring and longer term studies evaluating disease progression in the brain of NiV-MY-infected AGM, including in survivors, would be required to make this determination.

Recent work has suggested that the dynamics and timing of the adaptive immune response, specifically the humoral response, may play an important role in the response to infection in the AGM NiV-MY model [36]. Neutralizing antibody responses have typically been noted after PID 12 for NiV-MY-infected AGM [18, 36], including AGM 4 on the present study. However, animals which have strong neutralizing antibody responses still develop lesions in the brain, detected by magnetic resonance imaging and/or histologically, on or after PID 9 which progressively get more severe and consistently correlate with moderate to marked multifocal encephalitis in the brainstem and cerebellum [17, 18]. Collectively, this information suggests that a humoral response to NiV cannot mitigate neurologic disease once the virus has crossed the blood-brain barrier. The implications of this finding warrant further investigation. For instance, vaccine and drug development efforts should take into consideration that the virus could cross the blood-brain barrier and establish infection in the brain very rapidly after virus exposure. Therefore, studies related to these countermeasures should evaluate immune markers and correlate their appearance with the spread of the virus, particularly entry and establishment of infection in the brain. Since clinical disease manifestations for the AGM NiV-MY i.t. model are consistent and relevant to the human condition, and since it provides an avenue for the study of encephalitis outside of human symptomology and autopsy results, this model could be extremely important and relevant for future countermeasure efficacy studies.

While many aspects of human NiV disease can be replicated in the AGM model, much work still needs to be done to further understand certain features of this model. One such characteristic is the dynamics of virus entry into the CNS. Temporal-spatial assessments of virus entry into the CNS of AGMs have not been performed. In addition, the effect of exposure route on entry into the CNS has not been evaluated. For example, even though the virus may be able to enter the brain very rapidly following intranasal instillation (as could be predicted based on studies conducted in hamsters) [31], entry may be temporally delayed following i.t. inoculation where the virus is not immediately flushed over olfactory epithelial surfaces that allow rapid entry into the CNS (i.e. via the olfactory bulb). There may be even more significant impacts on entry dynamics following foodborne transmission, which is a highly prevalent mode of transmission during human outbreaks [1]. Understanding the rate of entry into the CNS is a critical gap that could have significant impacts on therapeutic interventions. If treatment is initiated after the virus enters the brain, the issue becomes the ability of the treatment to cross the blood-brain barrier and mitigate acute phase and chronic neurologic disease. However, without knowing when in the infection cycle the virus enters the CNS, there is currently no concrete way to gauge efficacy of medical countermeasures against the neurologic disease.

As discussed above, disease in the AGM model closely mimics the pathology in cases of human disease. However, there are subtle pathologic differences that may be identified. These differences are primarily in the lesion degree (i.e. severity) and less often in the presence or absence of a given lesion. As an example, while viral syncytia, viral labeling by ISH, inflammation and tubular epithelial necrosis are findings in this study in the kidney, lesions do not appear as severe as the degree of necrosis, thrombosis and vasculitis reported in the kidneys in humans [15]. Vasculitis in other organs such as the heart may also occur in humans with a higher degree of frequency [15], compared to what we observed in our study. Other differences include the presence of viral inclusion bodies (cytoplasmic and/or nuclear), which have been reported in cases of human infection, particularly in the CNS [15, 33], but were not seen in this study and have not been reported in a subset of the other studies utilizing this model [17–19]. Nonetheless, viral labeling and/or presence of viral antigen remains relatively consistent across Nipah studies utilizing this model and when compared to human disease. Utilization of electron microscopy may aid in more consistently identifying the presence/absence of viral inclusions in future Nipah virus studies. Overall, the NiV-MY i.t. AGM model represents a valuable tool for the study of NiV pathogenesis and countermeasure efficacy. Latent virus in the CNS with subsequent recrudescence is a major problem in NiV-infected patients, with development in up to 3.4% of patients that were originally asymptomatic [12]. Treatments that cannot affect the virus before it enters the brain or alter disease pathogenesis once it enters the brain will likely be of limited utility. It is, therefore, crucial that this NiV AGM model continues to be developed, promoting a further understanding of NiV pathogenesis which will be invaluable to countermeasure development in the future.

## Supporting information

S1 Fig Percent change in hematology parameters—Individual animal. The percent change from baseline was determined for each animal for the following hematology parameters: (A) white blood cells; (B) neutrophils; (C) eosinophils; (D) platelets. Shown in this figure are percent change over time for APN (black symbols) and CPN (red symbols).
(TIF)

S2 Fig Percent change in liver enzyme parameters—Individual animal. The percent change from baseline was determined for each animal for the following chemistry parameters: (A) ALT; (B) ALP; (C) AST; (D) GGT. Shown in this figure are percent change over time for APN (black symbols) and CPN (red symbols).
(TIF)

S3 Fig Percent change in serum chemistry parameters—Individual animal. The percent change from baseline was determined for each animal for the following chemistry parameters: (A) serum glucose; (B) amylase; (C) blood urea nitrogen; (D) creatinine. Shown in this figure are percent change over time for APN (black symbols) and CPN (red symbols).
(TIF)

S4 Fig GraphPad Prism file used to create manuscript graphs. File contains clinical pathology (hematology and clinical chemistry), temperature and qRT PCR data used to create graphs demonstrating changes in analyte values over the course of the study.
(PZF)

S5 Fig GraphPad Prism file used to create manuscript graphs. File contains graphs with multiple clinical pathology values grouped and displayed on the same graph.
(PZFX)
