# Supplementary figures and images for: Detailed analysis of the pathologic hallmarks of Nipah virus (Malaysia) disease in the African green monkey infected by the intratracheal route
Source: PLoS One. 2022 Feb 10;17(2):e0263834. doi: 10.1371/journal.pone.0263834 (PMC8830707; doi:10.1371/journal.pone.0263834)

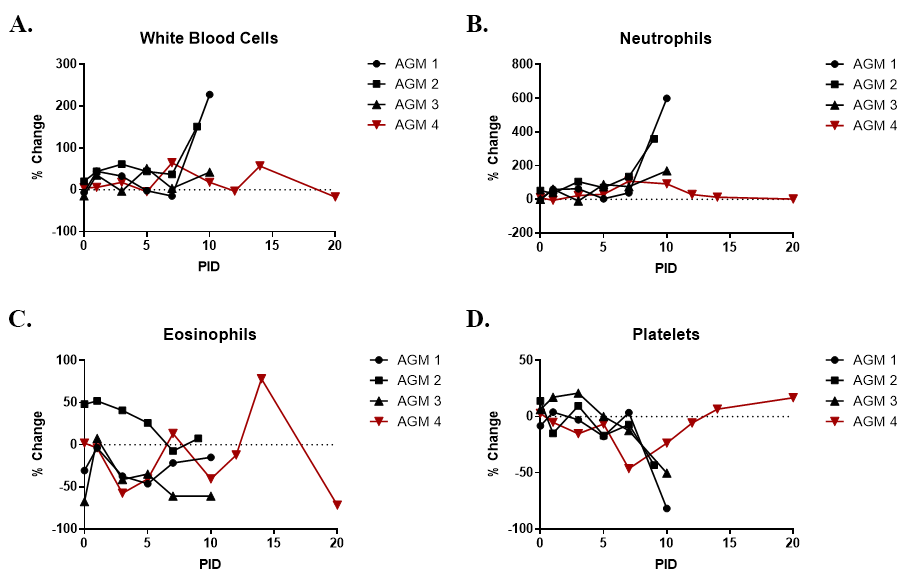

Supplement: S1 Fig — The percent change from baseline was determined for each animal for the following hematology parameters: (A) white blood cells; (B) neutrophils; (C) eosinophils; (D) platelets. Shown in this figure are percent change over time for APN (black symbols) and CPN (red symbols). (TIF) [file pone.0263834.s001.tif]

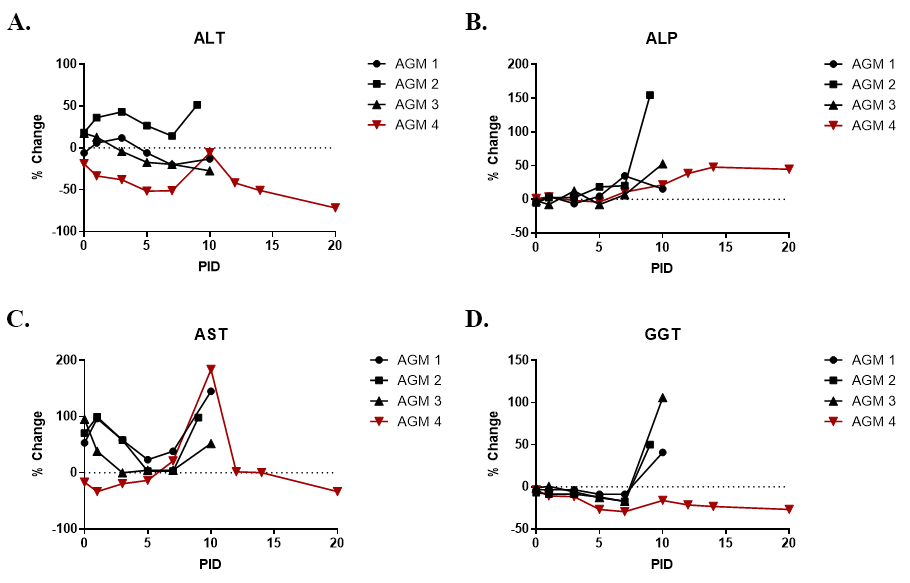

Supplement: S2 Fig — The percent change from baseline was determined for each animal for the following chemistry parameters: (A) ALT; (B) ALP; (C) AST; (D) GGT. Shown in this figure are percent change over time for APN (black symbols) and CPN (red symbols). (TIF) [file pone.0263834.s002.tif]

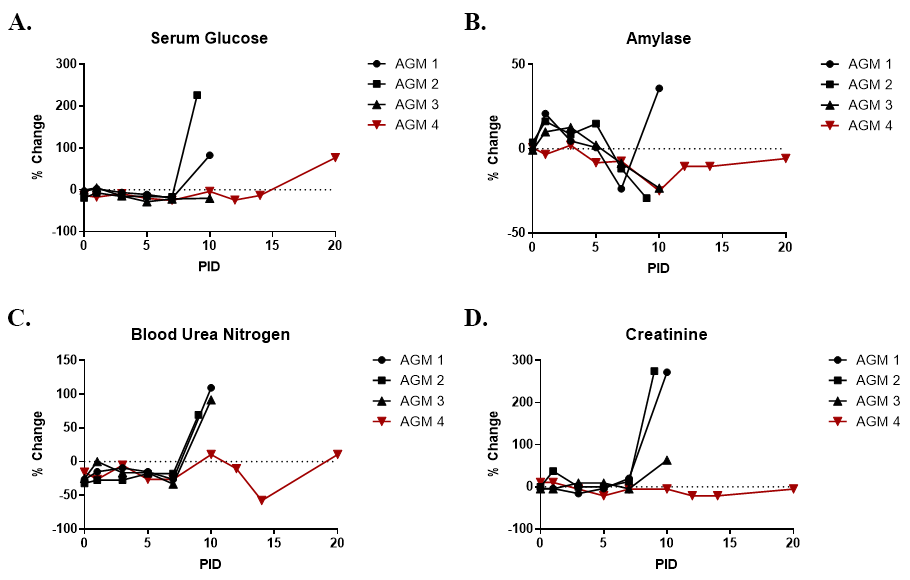

Supplement: S3 Fig — The percent change from baseline was determined for each animal for the following chemistry parameters: (A) serum glucose; (B) amylase; (C) blood urea nitrogen; (D) creatinine. Shown in this figure are percent change over time for APN (black symbols) and CPN (red symbols). (TIF) [file pone.0263834.s003.tif]
